# Platform Technology in Global Vaccine Regulation: Development, Applications, and Regulatory Strategies with Insights from China

**DOI:** 10.3390/vaccines12121436

**Published:** 2024-12-20

**Authors:** Xiaojing Li, Su Jin, Shuyang Guo, Dan Yang, Wenbo Sai, Xiao Qiu, Xin Zhao, Lan Wang, Tao Wang, Min Li

**Affiliations:** 1 Center for Drug Evaluation, National Medical Products Administration, Zone 2, No. 22 Guangde Street, Beijing Economic and Technological Development Zone, Beijing 100076, China; lixj@cde.org.cn (X.L.); jins@cde.org.cn (S.J.); guoshy@cde.org.cn (S.G.); yangdan@cde.org.cn (D.Y.); saiwb@cde.org.cn (W.S.); qiux@cde.org.cn (X.Q.); zhaoxin@cde.org.cn (X.Z.); 2 State Key Laboratory of Drug Regulatory Science, National Institutes for Food and Drug Control, Beijing 102629, China; wanglan@nifdc.org.cn

**Keywords:** platform technology, vaccine regulation, regulatory strategies, emergency vaccine approval, public health

## Abstract

The concept of “platform technology” gained prominence after the Ebola outbreak and since then has become essential to international vaccine (prophylactic vaccines against infectious disease) regulatory frameworks. Its significance was further amplified during the COVID-19 pandemic, where platform technology enabled the rapid development and approval of vaccines, optimizing regulatory processes, and enhancing global public health responses. As a transformative tool, platform technology streamlines product development, allowing for the reduction in the number of clinical trials or exemption from certain clinical trials and facilitating cross-referencing in regulatory submissions. Despite significant efforts to establish standardized regulatory procedures, challenges remain, particularly in achieving a unified definition and application of platform technology across regions. This paper explores the evolution, applications, and regulatory strategies of platform technology, with a focus on China’s experience in this field. China’s approach, encompassing risk assessment, and the expedited approval of emergency vaccines, offers valuable insights into global regulatory coordination. By analyzing China’s regulatory contributions and international practices, this paper highlights the potential of platform technology to address future pandemics, including “Pathogen X”, and underscores the importance of harmonizing global regulatory efforts to strengthen public health preparedness and response.

## 1. Introduction

Platform technology refers to a standardized and replicable approach for the development and production of similar biologics, including vaccines. This approach involves utilizing a well-characterized, identical product backbone—a single vector or expression system that serves as the core carrier in the manufacturing platform. This backbone acts as the foundational framework into which genes of interest are inserted through a standardized process, resulting in the generation of various recombinant master seeds, master sequences, or constructs, which are then used to produce the vaccine. A vaccine platform may be developed for certain vaccine types, such as nucleic acid vaccines containing DNA or mRNA, particular viral vectors or antigenic proteins with comparable antigenicity, amino acid composition and function in the pathogen it has been derived from. Importantly, the manufacturing process, critical quality attributes (CQAs), and quality control strategies can remain largely consistent, offering significant efficiencies in biologics development [1,2,3,4]. Over time, platform technology has evolved as an innovative approach or modular methodology, supported by regulatory agencies to encourage industry adoption. It provides a novel pathway to streamline and enhance regulatory processes through the integration of advanced technologies.

Vaccination, dating back to Edward Jenner’s pioneering work in 1796, is one of the most successful and cost-effective medical interventions in history. Today, despite the availability of approximately 27 licensed vaccines protecting against infectious diseases caused by over two dozen pathogens, many diseases remain without effective vaccines, presenting ongoing challenges for public health [5]. The COVID-19 pandemic further highlighted the need for rapid, adaptable vaccine solutions, positioning platform technology at the forefront of vaccine development [6].

The Ebola outbreak brought vaccine platform technology into the regulatory spotlight, where it gained significant traction as a means to accelerate the development and approval of urgently needed vaccines [7]. Although platform technology applications vary depending on the specific technical requirements of each field (Table 1), the underlying principle remains the same: it leverages a manufacturer’s accumulated expertise—production processes, product stability, method validation, quality specifications, product-specific batch release assays, and non-clinical and clinical data—to accelerate the development of new biologic candidates. By maintaining the core product backbone and modifying only the target gene sequence when necessary, platform technology serves as a key tool in streamlining regulatory processes and responding swiftly to emerging global health threats.

This paper aims to provide insights into the evolution, definitions, applications, and regulatory approach of platform technology in vaccine development, with a particular emphasis on China’s regulatory framework. It also highlights the broader impact of platform technology on global health, focusing not only on its role in the development of preventive vaccines against infectious disease and pandemic preparedness, but also its potential applications in routine immunization and therapeutic development of biologicals.

## 2. Application and Evaluation of Platform Technology

Platform technology has revolutionized the landscape of drug development by promoting innovation while significantly enhancing regulatory efficiency across the product lifecycle. With validated platform frameworks, the development process for new biologic candidates can be streamlined, reducing both time and costs under the premise of sufficient benefit-risk assessment and manageable risk. By minimizing redundant reviews, platform technology allows for rapid progression through regulatory pathways, addressing urgent clinical needs and improving access to innovative therapies [8]. During pandemics, this streamlined approach is especially crucial, as it enables timely responses to emerging pathogens, initiation of clinical trials, and regulatory authorizations based on real-world evidence from the original licensed vaccine product and accelerated regulatory frameworks [9].

The application of platform technology spans various stages of research and development (R&D) and regulatory processes, helping achieve different objectives across biological production development pipelines. From streamlining validation to simplifying submission requirements by allowing reference to previous regulatory submissions and/or authorizations, platform technology enhances efficiency and decision-making in regulatory approvals. These applications can be categorized as follows.

### 2.1. Streamlining Chemistry, Manufacturing, and Controls (CMCs) Validation and Research

Platform technology accelerates product development and iteration (updating vaccines to achieve broadly cross-reactive immune responses in the context of continued pathogen evolution) by providing comprehensive data from a previous vaccine product based on the same vaccine platform. This facilitates quicker assessments of products within the platform’s boundaries and enables faster development, particularly in areas such as viral clearance validation, and CMC changes [4,10]. Throughout the process, clinical development and availability of the market can be advanced with greater efficiency. For example, based on a thorough understanding of the platform quality attributes, process validation or evaluation can be appropriately reduced without compromising product safety. This streamlining research is a rational decision based on product and process knowledge, under the premise of ensuring reproducibility and consistently delivering safe and quality-controlled products [11].

### 2.2. Exemption of Non-Clinical/Clinical Data and Pre-Approval Inspection

The COVID-19 pandemic demonstrated the critical need for rapid vaccine development using platform technology. The International Coalition of Medicines Regulatory Authorities (ICMRA) and the WHO Technical Advisory Group on COVID-19 Vaccine Composition (WHO-CO-VAG) have emphasized that, by referencing an original licensed specific product based on the same platform technology of the same manufacturer can be envisaged for the approval of strain changes in currently authorized or approved COVID-19 vaccines from a given manufacturer, clinical trial data and pre-approval inspection for the specific adapted vaccine would be considered for reduction prior to approval of the recommended updated composition, provided no major changes to the manufacturing process or vaccine construct are introduced [12,13,14]. Post-authorization/approval clinical data may still be requested, as real-world evidence is critical for the ongoing evaluation of optimized dosage regimens, target populations, and vaccine strategies for variants, thereby supporting enhanced regulatory decision-making [15]. In this context, the use of platform technology combined with real-world evidence will play an integral role in the development, review, and iteration improvement in vaccines [16].

### 2.3. Simplified Submission Dossier Through Cross-Referencing

For new biologic candidates developed using an established platform, some well-validated chemistry, manufacturing, and controls (CMCs) data can be cross-referenced, thereby minimizing duplicate submissions during the regulatory process. While the requirements of leveraging platform technology information may vary in different scenarios, the criteria for overall product quality control and risk management are not compromised. In principle, regulatory evaluations focus on managing risk, guided by the maturity, durability, and representativeness of the platform for the particular product or process [17]. Early communication between applicants and regulatory authorities is encouraged to ensure that platform technology is appropriately established and applied.

In summary, platform technology offers a robust framework that accelerates biologics development, facilitates regulatory decision-making, and improves global health outcomes by providing a flexible, efficient, and risk-managed approach to addressing emerging public health threats.

## 3. Global Regulatory Processes and Technical Guidelines

### 3.1. Global Regulatory Advancements in Platform Technology

In recent years, regulatory authorities worldwide have made significant strides in establishing standardized regulations and procedures for the application of platform technology (Table 1). The European Medicines Agency (EMA) pioneered this effort by introducing concepts of platform technology and the vaccine platform technology master file (vPTMF) in the veterinary vaccine field in 2022 [2]. In 2023, under the European Commission’s pharmaceutical legislative proposals, the concept of master files was proposed to be extended from active chemical substances to a more general quality master file, including those for excipients, biological drug substances, and adjuvants. Building on this, the European Federation of Pharmaceutical Industries and Associations (EFPIA), Vaccines Europe (VE), and the Coalition for Epidemic Preparedness Innovations (CEPI) proposed amendments to further include platform technology master file (PTMF) and to clarify the legal definitions of platform technology [18]. In the United States, the importance of establishing a platform technology program to enhance regulatory processes and pandemic preparedness was emphasized within the 2022 Food and Drug Omnibus Reform Act (FDORA) and PREVENT pandemic Act. In response, the U.S. Food and Drug Administration (FDA) issued the “Platform Technology Designation Program for Drug Development Guidance for Industry” in May 2024, covering fields such as cell therapy, gene therapy, and mRNA vaccines, aiming to standardize and streamline the development of these advanced therapies incorporating designated platform technologies [3,8].

Although regulatory approaches became more specialized and differentiated across individual regulatory authorities, the regulatory platform approach began to emerge.

### 3.2. Challenges and Regional Variations in Establishing Regulatory Approach for Platform Technology

Despite these advancements, the systematic technical evaluation and mature supporting management system for platform technology remain underdeveloped. Regional variations in the application of this regulatory tool further complicate the establishment of a cohesive global regulatory framework. The formalization of a corresponding regulatory system is urgently needed, encompassing technical justification, synchronized management processes, and flexible dossier submission and inspection protocols.

The use of a master file (MF) for modular and flexible management can significantly contribute to process management and dataset utilization. Initially, Europe used the master files primarily for evaluating small molecule substances, vaccines, and blood products. However, the concept of platform technology master files has gradually been introduced, as seen in Annex II of Regulation (EU) 2019/6. By 2022, guidelines for veterinary vaccine platform technology master files were issued. The European Commission’s pharmaceutical legislation proposals, along with position papers from organizations such as EFPIA/VE/CEPI, have suggested expanding the master file concept to more fields, including vaccines, biological products, starting materials for antibody-drug conjugates (ADCs), and radiopharmaceuticals, as well as excipients, adjuvants, packaging materials, and components of drug-device combinations. This expansion aims to cover the entire product development and production lifecycle, facilitating cross-referencing of common adjuvants, common antigens, and more [19,20].

The promotion of the platform technology approach necessitates the simultaneous establishment of technical evaluation and management systems across various regions, combined with extensive global collaboration, to create a more harmonized regulatory framework. Regardless of the approach employed, the application of platform technology must comply with current regulations and guidelines. Manufacturers are required to demonstrate the significance and rationality for incorporating or using the platform technology and to ensure compliance in subsequent regulatory reviews. In the application of the platform approach, not all data can be cross-referenced for new products.

In summary, the evolution of global regulatory processes and technical guidelines for a regulatory platform approach is essential for advancing biological product development. By addressing regional challenges and fostering international collaboration, the regulatory landscape can be harmonized, ensuring the safe and efficient development of innovative therapies.

## 4. China’s Experience with Regulating Platform Technology

**Figure 1:**
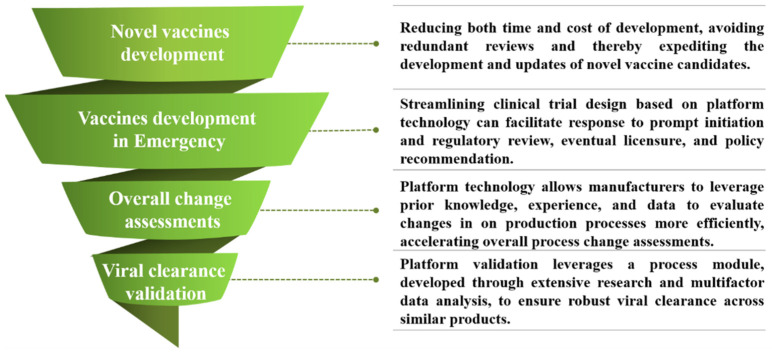
Regulatory Practices and Applications of a Regulatory Platform Approach in China. Platform approaches are data-driven and risk-based approaches that are integral to accelerating biological product development throughout the product lifecycle. This approach streamlines critical processes, facilitates process changes, and contributes to the overall product development to varying extents. It not only promotes biologics innovation but also enhances the efficiency of regulatory approval.

With the advancement and application of platform technology, Chinese regulatory authorities have progressively advanced the framework in response to its expanding role in vaccine development and biopharmaceutical innovations. As platform technology continues to evolve, Chinese regulators have introduced platform technology or platform validation in critical processes, such as viral clearance validation and process change during clinical trials (Figure 1). These efforts aim to provide clarity on the technical requirements and application of platform-based approaches in biological product development.

### 4.1. Viral Clearance Validation

China has made significant strides in optimizing viral clearance validation through the platform approach. The “Technical Guidelines for Virus Clearance Process Platform Validation of Therapeutic Recombinant Biotechnology Products at the CTA stage (Trial)” illustrate how platform validation can replace product-specific viral clearance validation for well-characterized recombinant protein products, such as monoclonal antibodies [10]. Platform validation leverages a process module, developed through extensive research and multifactor data analysis, to ensure effective and robust viral clearance across similar products. Typically, it is developed from sufficient case data or multi-factor orthogonal analysis, with the necessary sample size determined by the complexity and variability of process parameters. During platform validation, it is crucial to evaluate the representativeness and suitability of the process module. Key process parameters that affect viral clearance performance must remain within the worst-case conditions of the corresponding process module. Currently, China’s guidelines allow for the exemption of viral clearance validation for clinical study samples involving three or more products.

Compared to product-specific viral clearance validation, platform validation demands a higher level of technical expertise and knowledge on the side of the applicant. Manufacturers must integrate extensive product development experience with risk assessment strategies to ensure the safety and efficacy of products. Each application of platform validation requires a case-by-case analysis, emphasizing the importance of appropriate validation strategies and maintaining stringent regulatory standards [10].

### 4.2. Change Risk Assessment and Validation

Platform technology has become a valuable tool in managing and accessing risks associated with changes during product development. According to the “Guideline for Chemistry, Manufacturing, and Controls Changes to Biological Investigational Medicinal Products in Clinical Trials (Trial)” [4], “platform technology” or “platform knowledge” refers to “An existing technology, or group of technologies, applied to the development and/or production of products by a given manufacturer, whose previous products might have been marketed or received clinical trial approval. A platform would be considered to exist when the elements of the scaffold/skeleton (which may include nucleic acid vector, virus vector, protein backbone) or adjuvant (in conjunction with given antigens and vaccine types), the manufacturing processes, CQAs and GMP compliance are essentially unchanged. It can be regarded as a platform”. The experience and knowledge gained, e.g., the data generated from manufacturing, control and stability, method validation, and non-clinical and clinical data can all be used as supportive data for the more rapid assessment and development of a new candidate product that fits within the boundaries of the platform [4].

The platform approach helps assess risks associated with changes in product development. It allows manufacturers to leverage prior knowledge, experience, and data to evaluate changes in production processes more efficiently. This approach accelerates the evaluation of CMC changes and supports faster development of new products that fall within the boundaries of a particular established platform. This is particularly valuable during early clinical phases, where data may be limited, as platform technology provides a structured framework for managing CMC changes. By incorporating platform knowledge into risk assessment, manufacturers can make informed decisions that mitigate unforeseen risks. Additionally, as platform knowledge and data accumulate over time, the regulatory framework will mature, further enhancing the speed and quality of vaccine development while maintaining rigorous safety standards [21].

### 4.3. Development and Approval of Emergency Vaccines

The COVID-19 pandemic has highlighted the critical importance of the regulatory platform approach in responding quickly to emerging infectious diseases. The development of vaccines, such as mRNA, viral vector, and recombinant protein vaccines, demonstrates the ability of platform technology to facilitate rapid production through sequence replacement. Chinese guidelines, in alignment with international regulatory frameworks such as those from the FDA and EMA, streamline research and approval processes for vaccine development using platform technology [22].

In China, a development stage-specific strategy is applied throughout vaccine research and development, particularly for vaccines based on the same platform used for an already approved vaccine product against the same infectious disease. For instance, prototype vaccines with clinical study approval may have partially streamlined CMC studies in their subsequent iterations (updated vaccines that achieve broadly cross-reactive immune responses in response to the continued evolution of pathogens). Similarly, non-clinical studies for variant vaccines (a mutation-proof, next-generation vaccine to protect against upcoming variants) can be partially streamlined when the prototype vaccine has completed Phase II trials. Clinical trials may be simplified or reduced where applicable, under the premise that the prototype vaccine has finished Phase III trials. As more platform data accumulates, overall safety and effectiveness are continually validated, supporting expedited approvals during future pandemics without compromising vaccine safety and efficacy [16].

### 4.4. Regulatory Approaches for Conventional and Innovative Vaccines

Platform technology also offers promising opportunities for accelerating the development of conventional and innovative vaccines in China. In July 2023, the Center for Drug Evaluation (CDE) issued the “Technical Guidelines for Clinical Trials of Human Papillomavirus (HPV) Vaccines (Trial)”, which clarifies the clinical endpoints and immunogenicity testing requirements for iterative vaccines. According to the guideline, clinical trials for an iterative HPV vaccine with higher valency, developed using the platform technology of an authorized original vaccine, can be streamlined or expedited to some extent if the new product shares fundamentally the same or highly similar production equipment/facilities, manufacturing processes, in-process controls, and specifications—differing only in the HPV types covered. However, if major changes are introduced compared to the original vaccine, a case-by-case analysis is required, taking into account the nature of the changes and the degree of support provided by existing data from the original vaccine based on the same platform of the identical manufacturer [23].

Beyond HPV vaccines, platform technology is facilitating the development of more complex vaccines, such as multivalent vaccines based on innovative and scientific evidence, that surpass traditional vaccines in terms of valency and complexity. By leveraging platform knowledge, particularly in the production and control of polysaccharides and conjugates, Chinese regulatory authorities are supporting faster preclinical development and dynamic verification of these innovative vaccines [24].

A similar approach can be applied to mRNA vaccines in China using platform technology, with practical considerations taken into account. Applicants should explain and justify which parts of the platform technology are applicable, such as the construction of DNA templates for mRNA vaccines and the use of identical LNP lipid excipients. For critical factors affecting the active substance and product quality attributes—such as the total lipid to mRNA content ratio and kinetics of LNP assemble—adequate comparability studies are recommended.

In summary, China’s experience with the regulatory platform approach reflects a forward-thinking orientation that combines innovation with stringent regulatory oversight. By integrating platform-based strategies into its regulatory framework, China is enhancing its capacity to accelerate vaccine development, improve public health outcomes, and respond swiftly to future public health challenges.

## 5. Challenges in the Platform Regulatory Approach

The deployment and regulation of platform technology in vaccine development is shaped by a complex, multi-factor environment. Key considerations include differences in the maturity of platform technologies among manufacturers, the adoption of robust method development, and the extent to which these technologies have been validated across multiple products [25].

Additionally, product-related factors further complicate the regulatory process. These include variations in preventive vaccine product types, the characteristics of targeted infectious diseases (like Ebola and COVID-19), and the specific technical approaches employed (such as recombinant protein-based or nucleic acid vaccines). Each factor requires tailored regulatory scrutiny and careful consideration [26].

**Figure 2:**
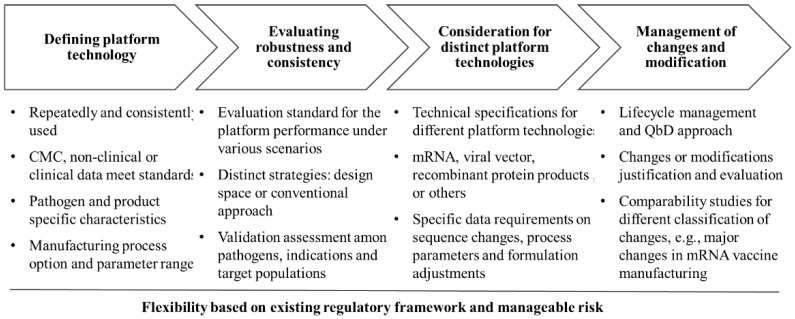
Challenges in the Platform Regulatory Approach. The complex, multi-factor challenges in implementing platform technology for vaccine regulation stem from the diversity of pathogens, differences in technical approaches, development strategies and performance robustness, the adequacy of clinical data, and change management.

In the context of China’s regulatory landscape, the implementation of platform technology in vaccine regulation faces several complex challenges, arising from both technical and regulatory factors. Below, we discuss key considerations for regulatory authorities, with particular insights drawn from China’s regulatory practices (Figure 2).

### 5.1. Defining Platform Technology

One of the primary challenges is establishing a clear and consistent definition of platform technology, which is essential for regulatory evaluation (Table 1). The definition of the term platform technology involves assessing its robustness and suitability based on the level of validation and supporting data, ultimately confirming the technology’s robustness. A platform qualifies as such if it can be repeatedly and consistently used for the production or validation of biologics, with the process performance, quality attributes, and non-clinical or clinical data of the product meeting established standards [27,28].

The flexibility in defining platform technology is contingent upon the existing regulatory framework, the understanding of products and processes, and the ability to assess and manage risks effectively. Typically, this requires consideration of factors such as product characteristics, pathogen characteristics, process mechanisms, and parameter ranges. For instance, a platform for a pandemic influenza vaccine can be defined after validating two mock-up pandemic vaccines and completing phase II clinical trials, whereas a COVID-19 vaccine platform necessitates Phase III efficacy data and safety information from over 3000 cases [29,30,31,32,33]. For conjugate vaccine technology platforms, marketing authorization of the product is the minimum requirement. Thus, the definition must be flexible enough to account for specific product characteristics while adhering to regulatory standards.

In the Chinese regulatory framework for platform technology used in preventive vaccines, a comprehensive understanding of quality attributes, assessments of pharmaceutical production, the accumulation of prior non-clinical and clinical data and other relevant factors should be integrated into the definition and consideration of platform technology, aligning with the regulatory approach.

### 5.2. Evaluating Robustness and Consistency

The robustness of the platform technology is critical to its ongoing use across product development. Regulatory authorities must establish standards to evaluate how well a platform accommodates changes, such as the introduction of new antigens or therapeutic targets. The challenge lies in maintaining consistent product characteristics, such as CQAs and clinical performance, across different applications. Variations in platform development strategies, pathogen characteristics, and intended populations introduce additional complexity. For example, companies may adopt varied strategies and foundations for platform technology development, such as traditional methods or more flexible “design space” approaches [34,35]. The scope of pathogens validated for each platform also varies, and the validation may span multiple pathogens and/or indication and usage, each requiring thorough assessment [36,37]. Additionally, clinical data obtained from populations during initial validation may differ significantly from those intended for future applications. For example, a platform originally validated for adults may be intended for pediatric use [24,38]. Regulators must assess the risks of such adaptations and ensure that platform robustness is maintained through comprehensive data review and comparability assessments.

### 5.3. Navigating Distinct Challenges of Different Technical Routes

In future pandemics, vaccine product development must prioritize both safety and efficacy while also ensuring rapid R&D and accessibility. Different platform technologies, such as mRNA or a specific viral vector vaccine, present distinct regulatory challenges due to their distinct safety profiles, development timelines, and responses to sequence changes. Each technical route requires tailored validation strategies, including process parameter adjustments, platform updates, and non-clinical studies, to evaluate the impact of changes on product quality and efficacy [39,40]. Therefore, specific technical specifications need to be formulated based on the chosen platform, including but not limited to the reconstruction and verification of engineered cells during sequence changes, process parameters and formulation adjustments, assessment of the impact on quality attributes, and the completion of necessary non-clinical studies.

For instance, mRNA vaccine platforms offer the advantage of rapidly incorporating new sequences, but the regulator’s oversight is needed to ensure consistent monitoring of CQAs [41,42]. It is recommended to establish a comprehensive database for different sequences and lengths and, where possible, to continuously collect data. This would enable thorough investigations into formulations, CQA characterization, RNA degradation characteristics, and stability of different sequences, thereby consistently strengthening platform validation and robustness. Alongside quality data, non-clinical and clinical data are crucial for validating platform technology, including studies on the biodistribution of mRNA-LNP, coding region translation, and the impact of degradation products on biological activity and clinical performance.

Viral vector vaccine platforms, on the other hand, may impose challenges in ensuring robustness when transitioning between different vector types. Different viral vaccine vectors belong to different platform technologies. Additional validation studies are required to confirm platform robustness. Replacing the antigen coding sequence within the same type of viral vector (such as Ad5) may have minimal impact on the product’s pharmaceutical characteristics. Adenoviral vaccine vectors derived from an identical adenovirus strain can serve as an effective platform technology following thorough validation, while the safety profiles and CQAs of different products are expected to obtain adequate justification, such as the absence of replication-competent adenovirus (RCA), purity and process- or product-related impurities. The robustness of the platform may be compromised when switching between different types of adenovirus vectors (e.g., Ad26), necessitating more evidence to validate the robustness of platform technology across various vector types [43].

### 5.4. Managing Changes and Updates to Platform Technology

Platform technology must be managed through a lifecycle management approach, with continuous improvements made as new data and insights emerge. Manufacturers are encouraged to adopt a Quality by Design framework, conducting extensive risk analysis and experimental designs early in platform development to anticipate future updates [44].

Platform technology continuously improves and advances through manufacturers’ enhanced understanding of products and processes, as well as the refinement of non-clinical or clinical data. Manufacturers must justify these changes in platform technology by evaluating their risk levels and impacts [45]. Ongoing updates to the platform’s supporting database are essential to maintaining its regulatory approval and ensuring it remains applicable to new products and therapeutic areas [17].

### 5.5. Leveraging Emerging Technologies to Address Regulatory Challenges

To tackle the challenges and enhance the regulatory landscape for platform technology, the integration of emerging technologies, such as high-throughput sequencing (HTS), artificial intelligence (AI), and organoid models, offers promising solutions. These tools can improve the precision of platform design and updates, particularly in areas like pathogen genomic surveillance, immunogenic epitope prediction, antigen design, infection dynamics, and disease modeling, as well as pharmacovigilance [46,47,48]. By incorporating these advancements, regulatory authorities and manufacturers can increase the efficiency of platform validation, accelerating biologics development while maintaining high standards of safety and efficacy [49].

In conclusion, while platform technology offers significant opportunities for accelerating vaccine development, itself and its regulatory approach present multifaceted challenges that require careful consideration of product-specific and technical factors.

## 6. Implications of Platform Technology for Pathogen X

### 6.1. Preparing for Future Pandemic Scenarios

The challenge in pandemic preparedness lies in acquiring the essential knowledge to promptly disseminate globally high-quality, cost-effective, and reliable countermeasures. It is essential to underscore the importance of employing platform technology strategies to bolster the capability to respond efficiently to unforeseen variants, emerging pathogens, zoonotic transmissions, and unknown threats such as “Pathogen X”. Pathogen X is envisioned as an unidentified future hazard that could originate from recognized viruses within each viral family or potentially from viruses that are currently unidentified, which may be often highly transmissible, have significant mortality, and tend to mutate rapidly. These characteristics necessitate rapid R&D and continuous updates and iterations in the short term [9].

For known pathogens, leveraging protection efficacy data derived from platform technology for immuno-bridging can effectively expedite research and development. This approach is especially applicable when the product is developed using the same technology platform and targets the same pathogen, potentially allowing for exemption from clinical efficacy trials once well-defined surrogate endpoints and reliable evaluation metrics are established [50,51].

When dealing with unknown pathogens, platform technology can be complemented by the use of representative antigen model strains from a related virus family. This approach involves extrapolating findings from prototype pathogens or model strains to actual circulating strains; however, its feasibility must be rigorously evaluated in relation to control requirements, pathogenesis (e.g., virulence, pathogen-host interactions), vaccine constructs and mechanisms of action, and the robustness of the platform technology, including existing clinical data. This combined strategy underscores the flexibility and adaptability of platform technology in pandemic preparedness [52].

### 6.2. Leveraging Platform Technology for Rapid Response: Challenges and Considerations

The potential of platform technology to enable rapid response during pandemics has been demonstrated, as seen in the 2022 Ebola outbreak, where vaccines against Sudan ebolavirus were granted for emergency use authorization within 9 weeks, leveraging data from the Zaire ebolavirus vaccine platform [53]. However, immuno-bridging between different pathogens requires careful consideration due to inherent differences in pathogenic characteristics, infection mechanisms, antigen structures, and immune responses [54].

Product-specific research is crucial to the limits and applicability of platform technology in cross-pathogen scenarios. Applicants are advised to carefully assess the extent to which platform-based data can support immuno-bridging and identify the specific conditions under which this strategy remains valid. Cross-platform immuno-bridging poses even greater challenges due to the complex nature of different vaccine mechanisms. This underscores the need for extensive international collaboration and research to explore and validate these mechanisms further [55].

Platform technology has profound implications for the rapid development and deployment of vaccines against Pathogen X and similar threats. By adopting a well-integrated regulatory strategy that leverages immuno-bridging, representative antigen models, and international cooperation, it is possible to enhance global pandemic preparedness and response capabilities while ensuring safety, efficacy, and adaptability across different platforms.

## 7. Conclusions and Future Directions

The lessons drawn from COVID-19 and Ebola underscore the importance of continued investment in basic, clinical, and implementation research, technology development, and engineering innovation [22]. Platform technology has played a pivotal role in accelerating global vaccine R&D, enhancing the precision of vaccine R&D across diverse approaches, and improving the efficiency of clinical trials and authorizations, serving as an effective strategy for combating Pathogen X and addressing future pandemics. Beyond pandemic preparedness, the integration of platform technology-based strategies in vaccine development offers substantial potential for driving innovation in other urgent clinical areas, contributing to the advancement of novel therapeutics and medical products [56].

Based on the broader impact of platform technology on global health, particularly its potential beyond pandemic preparedness such as in routine immunization and therapeutic biologicals development, more regulatory authorities worldwide have increasingly recognized the value of platform technology, even in non-pandemic contexts, particularly in areas such as biosimilars [8]. However, the application of platform technology in these situations requires careful deliberation. Different regulatory authorities may hold varying perspectives on its use, highlighting the importance of maintaining a balanced approach. Specific studies on platform-based products should be conducted, supported by a comprehensive risk-benefit assessment. The evaluation of platform technology’s applicability should consider factors such as platform type, product construct, clinical use and mechanism of action, processes and quality attributes, and the extent of clinical validation.

Although global institutions have made significant progress in exploring the regulatory platform approach as a scientific regulatory tool, challenges remain. The establishment of a benchmarking, systematic evaluation framework and supporting management processes is essential, particularly concerning platform definition, evaluation criteria, and change and update management. It is actionable to suggest programs and initiatives, e.g., fostering cross-border partnerships for the implementation of international guidelines or master files to ensure consistent standards across countries for platform technology or regulatory approach. International collaboration, led by agencies such as the WHO and CEPI, aims to tackle these challenges by coordinating efforts to develop a harmonized regulatory framework. As China continues to enforce regulatory standards related to platform technology, its guidelines, which cover aspects such as viral clearance, overall process change assessments, emergency, and conventional vaccine development, reflect a streamlined approach that adapts as platform-related data accumulates.

Looking ahead, the continued application of platform technology will be driven by ongoing advancements in productivity and regulatory science. Prospective research should focus on enhancing the comprehensive deployment of platform technology and synchronizing technological processes with regulatory management mechanisms. Technologically, it is crucial to establish a platform database built on well-defined principles of applicability, validation, and scenario-specific use. The lifecycle management of platforms should be guided by data-driven and risk assessment strategies, ensuring a robust and adaptable system.

In addition to technical justifications, the development of a synchronized regulatory system is urgently needed. This involves formalizing processes for defining platform technology, organizing and submitting dossier modules, and adjusting inspections and verifications practices. Expanding the use of the master file system to enable cross-referencing of common components may streamline process management and enhance data set utilization across different products.

The ultimate goal of these regulatory advancements is to improve the efficiency and predictability of biological product development while safeguarding safety, efficacy, and compliance with registration requirements. Drawing on the experiences gained from managing previous infectious disease responses, China will continue to refine its platform regulatory approach and regulatory systems. It is essential to justify the regulatory requirements deemed necessary, even when using a regulatory vaccine platform approach. This will facilitate the rapid, classified reviews of biological products adopting platform technology, whether in pandemic situations or under normal conditions, supporting the R&D and updating of innovative products across various fields. Meanwhile, efforts will be made to align with international regulatory trends, harmonize global policies for platform technology, and enhance global pandemic preparedness, contributing to the construction of a coordinated global public health governance.

## Figures and Tables

**Table 1:** Localized and International Guidelines for Platform Technology Application.

Guideline	Name	Definition	Platform Technology Instructional Description	Release Date
ICH Q11 Development and Manufacture of Drug Substances (Chemical Entities and Biotechnological/Biological Entities)	Platform manufacturing	Platform manufacturing is the approach of developing a production strategy for a new drug starting from manufacturing processes similar to those used by the same applicant to manufacture other drugs of the same type (e.g., as in the production of monoclonal antibodies using predefined host cell, cell culture, and purification processes, for which there already exists considerable experience).	A technical guideline to drug development that introduces the macro concept of platform manufacturing as a development tool to support process development	May 2012
ICH Q5A(R2) Viral Safety Evaluation of Biotechnology Products Derived from Cell Lines of Human or Animal Origin	Platform validation	Platform validation is defined as the use of prior knowledge, including in-house experience with viral reduction data from other products, to claim a reduction factor for a new similar product, according to current understanding.	A technical guideline for virus safety evaluation, which clarifies the concept and general principles of platform validation, and provides specific cases for different processes	November 2023
WHO TRS1011 Guidelines on the Quality, Safety and Efficacy of Ebola Vaccines	Platform technology	Platform technology is a production technology in which different viral vectored vaccines are produced by incorporating heterologous genes for different proteins into an identical viral vector backbone.	A technical guideline supports the development and licensing of an Ebola vaccine, which discusses opportunities to accelerate vaccine development and product availability based on “platform technology” during a public health emergency, the minimum dataset for CMC, non-clinical, and clinical research and evaluation required are highlighted.	July 2018
WHO TRS1039 Evaluation of the Quality, Safety and Efficacy of Messenger RNA Vaccines for the Prevention of Infectious Diseases: Regulatory Considerations	Platform technology	Platform technology is a group of technologies used as a base upon which other applications, processes or technologies are developed. In the context of mRNA vaccines, a given manufacturer might have one or more platforms on which they will develop vaccines (or therapeutics) against various diseases or pathogen strains.	A technical guideline for mRNA vaccines, which outlines prerequisites for platform technology in process development, stability, and clinical and non-clinical studies and explains the considerations for reducing non-clinical studies (abbreviated nonclinical program) under different circumstances and using immunobridging data to demonstrate protective efficacy during public health emergencies.	October 2021
EMA’s Guideline on Data Requirements for Vaccine Platform Technology Master Files (vPTMFs) for Veterinarians	Platform technology	In practice, a vaccine platform is a manufacturing process that relies on a single vector or expression system (“backbone carrier”) and a standard process for inserting a gene or genes of interest into the system to generate different recombinant master seeds, master sequences or constructs, which are then used to produce a vaccine. The gene of interest may consist of one or more complete or partial gene sequences. The active substance/s obtained is blended with adjuvants and/or excipients for the different target species to manufacture finished products with certain defined properties.	Guideline on data requirements for vPTMF, which outlines the data to be included in the vPTMF, as well as the data requirements for initial submission and re-evaluation.	January 2022
FDA Platform Technology Designation Program for Drug Development	Platform technology	Platform technology is a well-understood and reproducible technology, which can include a nucleic acid sequence, molecular structure, mechanism of action, delivery method, vector, or a combination of any such technologies that the Secretary determines to be appropriate, that the sponsor demonstrates (1) is incorporated in or used by a drug and is essential to the structure or function of such drug; (2) can be adapted for, incorporated into, or used by, more than one drug sharing common structural elements; and (3) facilitates the manufacture or development of more than one drug through a standardized production or manufacturing process or processes.	A procedural guide for platform technology, which describes the scope of platform technology designation, application time and procedure, etc.	May 2024
CDE, NMPA Technical Guidelines for Virus Clearance Process Platform Validation of Therapeutic Recombinant Biotechnology Products at the CTA stage (Trial)	Platform validation	Platform validation refers to a study method evaluating the virus clearance performance of specific process steps in the manufacturing process of other similar in-house products based on the internal experience of virus clearance process validation studies of similar products in combination with external knowledge appropriately.	A technical guideline for virus clearance process platform validation, including a clear description of how to establish a platform validation and the requirements for data establishment	January 2024
CDE, NMPA Guideline for Chemistry, Manufacturing, and Controls Changes to Biological Investigational Medicinal Products in Clinical Trials (Trial)	Platform technology	Platform technology is an existing technology, or group of technologies, applied to the development and/or production of similar products by a given manufacturer whose previous products might have been marketed or received clinical trial approval. A platform would be considered when the elements of the scaffold/skeleton (which may include nucleic acid vector, virus vector, protein backbone, etc.) or adjuvant, the manufacturing processes, CQAs and compliance GMP are essentially unchanged. It can be regarded as a platform. The experience and knowledge gained, data generated (manufacturing, control, and stability) and the method validation, non-clinical and clinical data, etc. can all be used as supportive data for the more rapid assessment and development of a new candidate product that fits within the boundaries of the platform.	Technical guidelines for CMC changes, which explain the scientific strategy and considerations for using platform technology to accelerate the development and evaluation of CMC changes for biological products in clinical trials.	June 2024

## Data Availability

No new data were created or analyzed in this study. Data sharing is not applicable to this article.

## References

[1] World Health Organization (2024). WHO TRS1039 Evaluation of the Quality, Safety and Efficacy of Messenger RNA Vaccines for the Prevention of Infectious Diseases: Regulatory Considerations.

[2] European Medicines Agency (2024). EMA’s Guideline on Data Requirements for Vaccine Platform Technology Master Files (vPTMF) for Veterinarians.

[3] U.S. Food and Drug Administration (2024). FDA Platform Technology Designation Program for Drug Development Guidance for Industry.

[4] Center for Drug Evaluation of NMPA (2024). Guideline for Chemistry, Manufacturing, and Controls Changes to Biological Investigational Medicinal Products in Clinical Trials (Trial).

[5] Rappuoli R., Alter G., Pulendran B. (2024). Transforming vaccinology. Cell.

[6] Farlow A., Torreele E., Gray G., Ruxrungtham K., Rees H., Prasad S., Gomez C., Sall A., Magalhães J., Olliaro P. (2023). The future of epidemic and pandemic vaccines to serve global public health needs. Vaccines.

[7] World Health Organization (2024). WHO TRS1011 Guidelines on the Quality, Safety and Efficacy of Ebola Vaccines.

[8] Niazi S.K. (2024). The United States Food and Drug Administration’s Platform Technology Designation to Expedite the Development of Drugs. Pharmaceutics.

[9] World Health Organization (2024). WHO Pathogens Prioritization: A Scientific Framework for Epidemic and Pandemic Research Preparedness.

[10] Center for Drug Evaluation of NMPA (2024). Technical Guidelines for Virus Clearance Process Platform Validation of Therapeutic Recombinant Biotechnology Products at the CTA Stage (Trial).

[11] O’Donnell K., Kartoglu U. (2024). QRM, Knowledge Management, and the Importance of ICH Q9(R1). Pharm. Technol..

[12] World Health Organization (2024). Statement on the Outcomes of the ICMRA-WHO Joint Workshop on COVID-19 Vaccines Strain Change.

[13] International Coalition of Medicines Regulatory Authorities (2024). Report from the ICMRA/WHO Workshop on: Global Perspectives on COVID-19 Vaccines Strain Update Alignment on Timing and Data Requirements.

[14] European Medicines Agency (2024). COVID-19 Vaccine Strain Updates: Global Regulators Agree on Timing and Data Requirements.

[15] Bollaerts K., Wyndham-Thomas C., Miller E., Izurieta H.S., Black S., Andrews N., Rubbrecht M., Van Heuverswyn F., Neels P. (2024). The Role of Real-World Evidence for Regulatory and Public Health Decision-Making for Accelerated Vaccine Deployment—A Meeting Report. Biologicals.

[16] Xu L., Li M., He W. (2024). COVID-19 Vaccine: Recent Advancements and Future Prospects. MedComm.

[17] Skritt J.H., Tucek-Szabo C., Sutton B., Nolan T. (2024). The Platform Technology Approach to mRNA Product Development and Regulation. Vaccines.

[18] European Federation of Pharmaceutical Industries and Associations (2024). Position on the European Health Emergency Preparedness and Response Authority (HERA).

[19] Francis M.J. (2022). Considerations for Rapid Development and Licensing of Conventional and Platform Technology Veterinary Vaccines. Avian Pathol..

[20] Entrican G., Francis M.J. (2022). Applications of Platform Technologies in Veterinary Vaccinology and the Benefits for One Health. Vaccine.

[21] International Council for Harmonisation of Technical Requirements for Pharmaceuticals for Human Use (2024). ICH Q11 Development and Manufacture of Drug Substances (Chemical Entities and Biotechnological/Biological Entities).

[22] Kalinke U., Barouch D.H., Rizzi R., Lagkadinou E., Türeci Ö., Pather S., Neels P. (2022). Clinical Development and Approval of COVID-19 Vaccines. Expert Rev. Vaccines.

[23] Center for Drug Evaluation of NMPA (2023). Technical Guidelines for Clinical Trials of Human Papillomavirus (HPV) Vaccines (Trial).

[24] Shirley M. (2023). 20Valent Pneumococcal Conjugate Vaccine: Pediatric First Approval. Pediatr. Drugs.

[25] Gupta A., Rudra A., Reed K., Langer R., Anderson D.G. (2024). Advanced Technologies for the Development of Infectious Disease Vaccines. Nat. Rev. Drug Discov..

[26] Heaton P.M. (2020). Challenges of Developing Novel Vaccines With Particular Global Health Importance. Front. Immunol..

[27] Daniel S., Kis Z., Kontoravdi C., Shah N. (2022). Quality by Design for enabling RNA platform production processes. Trends Biotechnol..

[28] Nag K., Sarker M.E.H., Kumar S., Khan H., Chakraborty S., Islam M.J., Baray J.C., Khan M.R., Mahmud A., Barman U. (2022). DoE-derived continuous and robust process for manufacturing of pharmaceutical-grade wide-range LNPs for RNA-vaccine/drug delivery. Sci. Rep..

[29] Ghattas M., Dwivedi G., Lavertu M., Alameh M.G. (2021). Vaccine Technologies and Platforms for Infectious Diseases: Current Progress, Challenges, and Opportunities. Vaccines.

[30] European Medicines Agency (2016). EMA Guideline on Influenza Vaccines Non-Clinical and Clinical Module.

[31] U.S. Food and Drug Administration (2023). Development and Licensure of Vaccines to Prevent COVID-19 Guidance for Industry.

[32] European Medicines Agency (2024). COVID-19: Lessons Learned from the Joint Report-Response to Public Health Emergency.

[33] European Medicines Agency (2024). Initiatives for Acceleration of Development, Support, and Evaluation Procedures for COVID-19 Treatments and Vaccines.

[34] Josefsberg J.O., Buckland B. (2012). Vaccine Process Technology. Biotechnol. Bioeng..

[35] Buckland B., Sanyal G., Ranheim T., Pollard D., Searles J.A., Behrens S., Pluschkell S., Josefsberg J., Roberts C.J. (2024). Vaccine Process Technology—A Decade of Progress. Biotechnol. Bioeng..

[36] Wang C., Zhang Y., Dong Y. (2021). Lipid Nanoparticle-mRNA Formulations for Therapeutic Applications. Acc. Chem. Res..

[37] Kheirvari M., Liu H., Tumban E. (2023). Virus-like Particle Vaccines and Platforms for Vaccine Development. Viruses.

[38] Syed Y.Y. (2023). Respiratory Syncytial Virus Prefusion F Subunit Vaccine: First Approval of a Maternal Vaccine to Protect Infants. Paediatr. Drugs.

[39] Monslow M.A., Elbashir S., Sullivan N.L., Thiriot D.S., Ahl P., Smith J., Miller E., Cook J., Cosmi S., Thoryk E. (2020). Immunogenicity Generated by mRNA Vaccine Encoding VZV gE Antigen is Comparable to Adjuvanted Subunit Vaccine and Better Than Live Attenuated Vaccine in Nonhuman Primates. Vaccine.

[40] Rai C.I., Kuo T.H., Chen Y.C. (2024). Novel Administration Routes, Delivery Vectors, and Application of Vaccines Based on Biotechnologies: A Review. Vaccines.

[41] Pardi N., Krammer F. (2024). mRNA Vaccines for Infectious Diseases—Advances, Challenges and Opportunities. Nat. Rev. Drug Discov..

[42] Cheng F., Wang Y., Bai Y., Liang Z., Mao Q., Liu D., Wu X., Xu M. (2023). Research Advances on the Stability of mRNA Vaccines. Viruses.

[43] Elkashif A., Alhashimi M., Sayedahmed E.E., Sambhara S., Mittal S.K. (2021). Adenoviral Vector-Based Platforms for Developing Effective Vaccines to Combat Respiratory Viral Infections. Clin. Transl. Immunol..

[44] Ampa C., Clenet D., Halpern J., Le Palaire L., Krishna M., McGoldrick M., Bilanin M., Pattnaik P., Pelt R., Restrepo S. (2024). Case Studies on Changes and Proposed Process Development Approaches Reflecting Applicability of PDA Technical Report No. 89 Strategies for Vaccine Development and Lifecycle Management. PDA J. Pharm. Sci. Technol..

[45] Lipa M.J., Greene A., Calnan N. (2021). Knowledge Management as a Pharmaceutical Quality System Enabler: How Enhanced Knowledge Transfer Can Help Close the ICH Q10 to ICH Q12 Gap. PDA J. Pharm. Sci. Technol..

[46] Barone P.W., Keumurian F.J., Neufeld C., Koenigsberg A., Kiss R., Leung J., Wiebe M., Ait-Belkacem R., Tabrizi C.A., Barbirato C. (2023). Historical Evaluation of the In Vivo Adventitious Virus Test and Its Potential for Replacement with Next Generation Sequencing (NGS). Biologicals.

[47] Vignali V., Hines P.A., Cruz A.G., Ziętek B., Herold R. (2022). Health Horizons: Future Trends and Technologies from the European Medicines Agency’s Horizon Scanning Collaborations. Front. Med..

[48] Wang H., Brown P.C., Chow E.C.Y., Ewart L., Ferguson S.S., Fitzpatrick S., Freedman B.S., Guo G.L., Hedrich W., Heyward S. (2021). 3D Cell Culture Models: Drug Pharmacokinetics, Safety Assessment, and Regulatory Consideration. Clin. Transl. Sci..

[49] Castellanos M.M., Gressard H., Li X., Magagnoli C., Moriconi A., Stranges D., Strodiot L., Tello Soto M., Zwierzyna M., Campa C. (2023). CMC Strategies and Advanced Technologies for Vaccine Development to Boost Acceleration and Pandemic Preparedness. Vaccines.

[50] King D.F., Groves H., Weller C., Jones I., Cramer J.P., Gilbert P.B., Goldblatt D., Gruber M.F., Kampmann B., Maïga D. (2024). Realising the Potential of Correlates of Protection for Vaccine Development, Licensure and Use: Short Summary. NPJ Vaccines.

[51] Huang Z., He J., Su J., Ou Z., Liu G., Fu R., Shou Q., Zheng M., Group T., Luxembourg A. (2021). Immunogenicity and safety of the quadrivalent human papillomavirus vaccine in Chinese females aged 9 to 26 years: A phase 3, open-label, immunobridging study. Vaccine.

[52] Cassetti M.C., Pierson T.C., Patterson L.J., Bok K., DeRocco A.J., Deschamps A.M., Graham B.S., Erbelding E.J., Fauci A.S. (2023). Prototype Pathogen Approach for Vaccine and Monoclonal Antibody Development: A Critical Component of the NIAID Plan for Pandemic Preparedness. J. Infect. Dis..

[53] World Health Organization (2022). Ebola Trial Candidate Vaccines Arrive in Uganda in Record 79 Days After Outbreak Declared.

[54] Albakri K., Al-Hajali M., Saleh O., Alkhalil A.M., Mohd A.B., Samain C.A., Abuasad N.N., Hasan H., Khaity A., Farahat R.A. (2023). Marburg Virus Disease Treatments and Vaccines: Recent Gaps and Implications. Ann. Med. Surg..

[55] Rousculp M.D., Hollis K., Ziemiecki R., Odom D., Marchese A.M., Montazeri M., Odak S., Jackson L., Beyhaghi H., Toback S. (2024). Reactogenicity Differences between Adjuvanted, Protein-Based and Messenger Ribonucleic Acid (mRNA)-Based COVID-19 Vaccines. Vaccines.

[56] Gebre M.S., Brito L.A., Tostanoski L.H., Edwards D.K., Carfi A., Barouch D.H. (2021). Novel approaches for vaccine development. Cell.

